# The relationship between peer learning and self-directed learning: the mediating role of collaborative leadership among pre-service mathematics teachers

**DOI:** 10.3389/fpsyg.2025.1724725

**Published:** 2026-01-13

**Authors:** Mustafa Çağrı Gürbüz, Ramazan Özkul, Sedat Şen

**Affiliations:** Faculty of Education, Harran University, Şanlıurfa, Türkiye

**Keywords:** collaborative leadership, peer learning, pre-service mathematics teachers, self-directed learning, structural equation modeling

## Abstract

**Introduction:**

This study aimed to examine the relationship between peer learning and selfdirected learning, as well as the mediating role of collaborative leadership among pre-service mathematics teachers.

**Methods:**

A cross-sectional quantitative research design was used, and data were collected from a state university in southeastern Türkiye. Structural equation modeling was used to estimate the structural relationships among peer learning, self-directed learning, and collaborative leadership in a group of pre-service teachers.

**Results:**

The results indicated a statistically significant positive correlation between collaborative leadership and both self-directed learning and peer learning. The relationship between peer learning and self-directed learning was negligible (*β* = −0.016, small effect) and not statistically significant, the relationship between peer learning and collaborative leadership (*β* = 0.776, large effect), and the relationship between collaborative leadership and self-directed learning (*β* = 0.777, large effect) were statistically significant. Moreover, gender, as a control variable, was determined to be non-significant, but estimates of grade level and project experience were identified as statistically significant and positive.

**Discussion:**

This study examines collaborative leadership as a mediator between peer learning and self-directed learning among Turkish pre-service teachers, representing a notable contribution to the literature. Self-directed learning is influenced by multiple factors, while peer tutoring enhances students’ engagement with their environment, cognitive growth, and social connection. Collaborative leadership has been identified as a significant mediating variable in this relationship. These results underscore the relevance of collaborative leadership as an important mechanism through which peer learning and self-directed learning are related. The findings suggest the potential effectiveness of establishing a project-based learning environment for pre-service teachers that focuses on fostering peer interaction and leadership development.

## 1 Introduction

Innovative approaches in education are increasingly gaining importance to support the professional development of pre-service teachers and improve teaching processes. One of the primary means of fulfilling this expectation is through the implementation of effective teaching-learning activities, which can be facilitated by innovative education programs (Salmi, 2009). However, higher education institutions continue to face criticism for the ineffectiveness of these activities (Burns and Köster, 2016; Leveson, 2000). At this juncture, peer learning (PL) and self-directed learning (SDL) emerge as efficacious methodologies for cultivating pre-service teachers’ knowledge and competencies (Lee and Bonk, 2024; Wu, 2021). PL has been shown to encourage students’ active participation in the teaching and learning process (King, 2002), while SDL allows individuals to develop their own learning skills (Penalva, 2023). The integration of these two learning approaches has the potential to enhance the effectiveness of pre-service teachers in the domain of mathematics education. However, in understanding this interaction, it is necessary to explain the existence of collaborative leadership (CL), which is considered a mediating variable. CL is a leadership model that encourages followers to work together to achieve a common goal and learn (Orchard et al., 2020). Núñez-Andrés et al. (2022) specifically emphasized that CL improves university-level learning outcomes and fosters higher-level skills. Yang (2021) demonstrated that CL enhances cognitive and social outcomes by strengthening group learning dynamics. Priest and Paula (2016) have demonstrated that the interaction and joint problem-solving processes of PL align with the fundamental principles of CL. Zhang and Bartol (2010) assert that CL positively impacts learning processes by enhancing the levels of knowledge sharing and interaction. While the existent literature highlights the positive aspects of CL, it has also been emphasized that further research is needed to determine the conditions under which it exerts a stronger or weaker influence on the behavior of team members (Liu et al., 2014). Burke et al. (2011) assert that it is essential for leadership studies to prioritize the process. Analyzing its impact on diverse factors (Edmondson, 1999) would guarantee enhanced consistency. The aforementioned points lend support to the proposition that CL functions as a mediating variable. Numerous studies highlight the academic benefits of structured peer feedback in the learning process (Double et al., 2020). Peer feedback has garnered attention for its contribution to fostering collaborative learning and enhancing academic performance (Dong et al., 2023; Wu and Schunn, 2023). Studies examining peer feedback in project-based collaborative learning environments have expressed the need for further empirical studies (Hadyaoui and Cheniti-Belcadhi, 2025). Collaborative learning is a pedagogical approach in which students work interdependently in order to construct knowledge. It has been determined that educators prioritize instruction, curriculum design, team composition, and evaluation factors when investigating teamwork pedagogy (Riebe et al., 2016). Different from collaborative learning, according to Crosby and Bryson (2005) and Orchard et al. (2020), CL is a shared leadership process in which members of a group jointly influence decisions, coordinate responsibilities, and work towards common goals. The present study focuses exclusively on CL as a leadership mechanism surfacing in team-based project settings. Thus, all theoretical explanations, measures, and structural relationships from this research pertain to leadership processes, not learning techniques. Researchers consistently express the need for empirical research to uncover possible interactions. While the literature strongly documents the benefits of PL, CL and SDL in teacher education, there is a notable gap in understanding the mechanisms and contextual factors that shape these relationships, particularly in non-Western, culturally diverse settings such as Türkiye. In this context, the strategies and practices necessary for pre-service teachers to become more effective and competent in teaching mathematics will be discussed. The fundamental process of this research was conducted within the scope of a project preparation course. Pre-service mathematics teachers were asked to form groups and become stakeholders in the project process for preparation purposes. This process created a natural collaborative and peer-learning environment. Assessing PL, CL, and SDL processes within a project-based learning framework offers a tangible benefit (Rivadeneira and Inga, 2023).

From a theoretical perspective, the relationship between PL and SDL may not be adequately explained as a direct or linear process. While PL creates interactive and cognitive opportunities, previous research suggests its effectiveness depends on how responsibility, influence, and decision-making processes are structured and shared within learning groups (Johnson and Johnson, 2009; Topping et al., 2017). Building on social constructivist theory of learning (Vygotsky, 1978) and self-regulated learning theory (Zimmerman and Schunk, 2011), which emphasizes the active role of CL in managing learning, this study argues that CL functions as a critical mechanism in transforming peer interactions into SDL behaviors. This research positions CL as a processual mediator that influences the emergence of autonomy, shared responsibility, and regulation during peer learning experiences, in contrast to studies that view leadership primarily as a consequence or contextual condition of collaboration (Orchard et al., 2020). In this sense, leadership is considered a dynamic and distributed process that organizes interaction and enables learners to assume collective and individual responsibility for learning (Pearce and Conger, 2003; van Schaik et al., 2014). By adopting this perspective, our study focuses not only on explaining whether PL is related to SDL but also on expanding existing theoretical frameworks by explaining how and under what conditions this relationship is realized in collaborative, project-based learning environments.

Building on the theoretical arguments outlined above, the present study aims to examine how PL, CL and SDL are systematically related within a project-based learning context. Specifically, PL is conceptualized as a key antecedent that provides the interactional conditions for learning, while SDL is treated as an outcome reflecting pre-service teachers’ capacity to regulate, sustain, and take responsibility for their learning. CL is positioned as a mediating process that structures how responsibility, influence, and decision-making are enacted within peer groups. Accordingly, the primary objective of this study is to investigate whether and how CL explains the relationship between PL and SDL among pre-service mathematics teachers. In addition, the study examines the extent to which selected individual characteristics (gender, grade level, and project experience) influence these relationships.

This study aims to examine whether and how CL is associated with the relationship between PL and SDL. Specifically, this research conducted on pre-service mathematics teachers aims to reveal the role of CL in PL and SDL processes and the contribution of these processes to the professional development of pre-service teachers. The research questions guiding the current study are

1. To what extent is pre-service teachers’ PL related to CL and SDL?
2. Does pre-service teachers’ CL mediate the relationship between PL and SDL?
3. Do pre-service teachers’ gender, grade level, and project participation status, as covariates, influence the relationships among PL, CL, and SDL?

## 2 Theoretical foundation

### 2.1 Peer learning

The definition of PL has been explained through the literature as having a history, where the origin of peer-supported learning dates back to Ancient Greek (Topping, 2005). In the preceding four decades, PL has become a prevalent teaching method (Keerthirathne, 2020). PL occurs among individuals from similar social groups, who in turn assist each other in the process of learning and facilitate it (Gogus, 2012). The PL process is defined as the facilitation of student-centered knowledge construction, rather than the passive absorption of knowledge, which is characteristic of traditional learning models (Kalem and Fer, 2003). By the conclusion of the educational program, students may have attained a level of proficiency in a specific task that would previously have been unattainable on an individual basis. Some studies have found that students, especially those learning through collaborative methodologies, tend to rate fellow students as potentially more accessible sources of information than instructors (Roberts, 2008). PL, a student-centered approach grounded in the principles of social constructivism (Palincasar and Herrenhohl, 1999), offers educators and students the opportunity to explore novel roles and underscores the human essence of education through personal interactions (Falchikov, 2003). Vygotsky (1978) posits that by emphasizing students’ interactions with their environment, it enhances the social interaction aspect of cognitive development. The concept of the “zone of proximal development” posits that learning occurs in a social environment, in accordance with the interests of the student, and with the guidance of more competent guides (Hogan et al., 1999). Peer learning is not solely based on social constructivist theory but also represents a multi-theoretical construct explained through social cognitive theory (Bandura, 1997), social interdependence theory (Johnson and Johnson, 2009), and self-regulated learning perspectives (Zimmerman and Schunk, 2011). The current study centers on project-based group work and leadership processes that emerge from peer interaction, utilizing social constructivism as the primary theoretical framework while also acknowledging supplementary perspectives. The fundamental tenets of this pedagogical approach encompass academic development, social interaction, enhanced self-efficacy and self-confidence, collaboration and a sense of community, role sharing (in both teacher and learner capacities), feedback exchange, and self-assessment (Miller et al., 2010; Wang, 2024). Moreover, PL has been shown to build trust, communication, and social ties among students, which makes them more interested and motivated to learn (Miller et al., 2010; Wang, 2024). Consequently, PL is a multifaceted approach that supports both academic achievement and social–emotional development. PL is becoming a more and more important part of math classes (Hidayat et al., 2023) and has been shown to make people feel better about math (Hidayat et al., 2023). The benefits of PL in the educational process include motivating students by supporting social participation (Fuchs et al., 1997) and providing social status among peers (Rohrbeck et al., 2003), sharing information, collaboration, leadership, and self-assessment in the project preparation process (Dosoftei and Alexa, 2024; Dutta et al., 2023; Shishkina, 2025; Torres et al., 2015). Peer interaction also has a positive effect on students’ positive attitudes toward mathematics (Steinberg et al., 1992) and academic motivation (Light and Littleton, 1999). Taken together, these studies suggest that peer learning is not merely an instructional technique but a social process that creates the conditions for shared responsibility and mutual influence—conditions that require leadership dynamics to be effectively translated into deeper learning outcomes.

### 2.2 Self-directed learning

SDL is defined as the process in which an individual assumes responsibility for managing, designing, and evaluating their learning process (Knowles, 1975). Brockett and Donaghy (2011) stated that SDL encompasses both individual responsibility and openness to social interaction. Furthermore, SDL serves different purposes, such as promoting an individual’s transformative role in the learning process and encouraging prosocial behavior (Brookfield, 2009). According to Long (2000), the concept of SDL encompasses three distinct psychological characteristics: self-regulatory ability, motivation, and metacognitive awareness. Self-management and self-regulation similarly emphasize active participation and goal-oriented behavior, are based on goal setting and task analysis, involve self-assessment in the learning process, and are classified as metacognitive skills (Loyens et al., 2008; Saks and Leijen, 2014). There are also differences between the two concepts. For example, SDL is used to describe a process in which a student is responsible for their own learning, while self-regulation is viewed as a student characteristic. There are numerous studies on SDL designed to measure the psychometric properties of individuals (Fisher et al., 2001; Williamson, 2007). The topic has been extensively investigated in the literature and has consequently been the focus of meta-analyses (Morris et al., 2025). SDL is increasingly associated with competency-based education, a framework that allows graduates to fulfill labor market demands using unconventional higher education methods (Özkul, 2025). In the present study, SDL is approached as a theoretically grounded construct that has been examined across a range of educational contexts (Knowles, 1975; Zimmerman and Schunk, 2011). This research does not confine SDL to a study-specific concept; instead, it utilizes established theoretical frameworks to investigate its manifestation in a specific instructional context—specifically, project-based PL among pre-service mathematics teachers. This viewpoint enables the study to enhance current dialogues within the SDL literature, acknowledging that the expression of SDL may differ across various contexts. These theoretical and empirical perspectives indicate that SDL does not develop in isolation but is shaped by social contexts that support autonomy, feedback, and shared regulation, leading to the need to examine social mechanisms that enable this process.

### 2.3 Collaborative leadership

CL is a leadership process in which influence, authority, and responsibility are shared among members of a group to achieve some common purpose. Rather than a pedagogical learning approach, CL emphasizes distributed decision-making, interdependence, coordinated action, mutual trust, and shared ownership of outcomes (Orchard et al., 2020; Orchard and Rykhoff, 2015). Educational contexts manifest CL when students collaboratively guide group processes, negotiate roles, and collectively enact leadership behaviors during project-based or team-based tasks. Such leadership dynamics shape how groups operate, resolve issues, and handle responsibilities. The literature has focused more on shared leadership than CL within teams and is most frequently associated with project work (Orchard et al., 2020). Discussions about collaborative or shared leadership also have a broader focus on art (Kramer and Crespy, 2011); it is evident in a variety of disciplinary fields, including business (Archer and Cameron, 2009), psychology (Bligh et al., 2006; Drescher et al., 2014), education (Morrison and Arthur, 2013), and military studies (Lindsay et al., 2011). In these disciplines, shared leadership processes have brought CL to the forefront in the context of team effectiveness (Orchard et al., 2020). In general, CL can be defined as a leadership approach in which individuals come together towards a common goal and act together to achieve an agreed-upon outcome. This approach aims to achieve success through the direction and coordination of the leader, based on the collective contributions of group members. In the field of education, this leadership style is characterized by the student taking on the role of an active participant in a collaborative team (Harmon and Johnson, 2024). Interdisciplinary CL is defined as an innovative educational approach that enables individuals from different disciplines to learn and problem-solve together by combining their knowledge, skills, and perspectives for a common goal (Clements and Lange, 2020; Lorente et al., 2024; Vogler et al., 2018). This process allows students to develop new knowledge and understanding in different fields, supporting the development of 21st-century skills such as communication, teamwork, and creativity (Clements and Lange, 2020; Gardiner, 2020; Vogler et al., 2018). A sense of trust, effective decision-making, and collaborative relationships among team members are critical for students to be successful in leadership (Orchard and Rykhoff, 2015). In this respect, CL is seen as a dynamic leadership type that integrates mutual relationships, information sharing, and effective decision-making (van Schaik et al., 2014). Accordingly, CL is best understood not as a static role distribution but as a dynamic process that structures interaction, responsibility, and decision-making within learning groups, thereby influencing how learning activities are enacted.

### 2.4 Collaborative leadership and peer learning

The reason why CL is considered one of the most common learning strategies in the literature is that it contributes to academic success, as well as increasing motivation and psychological support for individuals (Johnson and Johnson, 2009; Takahashi, 2011). Collaborative learning develops students’ high-level comprehension and retention skills (Bahar-Özvariş et al., 2006; Peklaj and Vodopivec, 1999). CL prioritizes sharing among group members rather than concentrating leadership on specific individuals. Bellibaş et al. (2025) state that shared and collaborative leadership supports the transformational aspect of education. Ertem (2021) and Göktaş and Kaya (2022) stated in their studies that CL increases participation and shared responsibility in decision-making processes within the organization, while Veyisoğlu and Bostancı (2024) stated that it creates synergy. PL is defined as a structured process through student interaction in which knowledge and skills are acquired (Topping et al., 2017). Li (2025) found that peer support significantly positively impacted collaborative learning activities and student engagement. It provides deep learning by encouraging active participation and responsibility among students, especially in higher education. In a systematic review of 172 studies, Markowski et al. (2021) concluded that PL improves students’ self-confidence and teamwork skills. The fundamental principles of CL also relate to PL’s emphasis on interaction and collaborative problem-solving processes. Research in project-based learning environments underscores the influence of shared leadership on students’ self-regulation, motivation, and group collaboration (Yilmaz et al., 2020). For the impact of PL to manifest, it is essential for leadership to be distributed and for students to engage in leadership roles (Lerchenfeldt et al., 2019). Similarly, the development of CL is made possible by participants’ active engagement in active learning and mutual support processes. In addition to these theoretical explanations, empirical studies support the relationship between these two variables: Alkhawaldeh and Khasawneh (2023), in their study investigating the role of CL in promoting peer interaction, show a statistically significant and strongly positive relationship between these two variables (*r* = 0.72). This suggests that PL processes are more effective and efficient in learning environments with high levels of CL, thus supporting collective learning dynamics.

### 2.5 Peer learning and self-directed learning

PL and SDL are closely related concepts and are expected to improve student learning. Social cognitive theory suggests that learning is more effective when students interact with peers who are similar to or have higher levels of self-efficacy (Bandura, 1997). It has been stated that students with low self-efficacy in SDL can strengthen their SDL skills by learning from more competent peers (Lee and Mori, 2020). Essentially, peer learning allows students to learn by developing their SDL skills (Zimmerman and Schunk, 2011). How PL facilitates SDL can be understood through several key components: providing more opportunities for student–student interaction, which provides greater learning opportunities than teacher-student interactions, and encouraging students to take responsibility for their learning. Shared responsibility plays a key role in PL. This role allows students to participate more effectively by sharing cognitive tasks together during interactions. This collaborative approach requires students to regulate their learning. Peer interactions increase students’ ability to independently direct their own learning by enabling them to self-assess (Sinkkonen and Tapani, 2024). Recent empirical studies in the literature support these findings: Lim et al. (2020) found that students’ PL skills had a positive and significant effect on academic achievement (*β* = 0.478) and significantly influenced students’ SDL strategies (*β* = 0.793), while Lee and Mori (2020) showed that PL improved SDL and helped students revise their learning approaches and learn new strategies from their peers. A regression analysis study conducted with pre-service teachers using information and communication technology revealed that PL contributed to SDL at a statistically significant level (*β* = 0.057) (Onivehu et al., 2018). PL is an important process in the development of SDL skills; students acquire and develop these skills through mutual exchange, collaboration, and self-assessment.

### 2.6 Collaborative leadership and self-directed learning

CL is an effective argument in student-centered learning environments that enable students to develop self-directed and collaborative learning skills (Hassan et al., 2024; Hutasuhut et al., 2021). This type of education encourages individuals to be both practitioners and active designers of the learning process. Its collaborative aspect provides students with opportunities for self-expression and also develops critical thinking skills (Avolio et al., 2009). On the other hand, SDL stands out as an approach in which individuals identify their learning needs, develop strategies, and take responsibility for the learning process (Knowles, 1975). CL can support students’ SDL skills by providing a socially and psychologically strong environment. Literature demonstrates that learning-focused learning has positive effects on SDL. The collaborative self-directed learning model enables students to take responsibility for their learning and apply knowledge in a practical way (Kemp et al., 2022). In this context, leaders enhance learning processes, thereby augmenting stakeholders’ SDL competencies and fostering collective learning capacity (Lundqvist et al., 2022). A longitudinal study demonstrated that learning-focused leadership enhances teachers’ dedication to lifelong learning via self-directed learning processes (Hassan et al., 2024). This finding suggests that CL contributes to teachers’ continuous professional development. This theoretical framework suggests that CL encourages individuals to take greater responsibility for their learning processes and makes learning environments more effective.

### 2.7 Conceptual framework

Our research literature indicates that the connection between peer interaction and SDL has been emphasized but insufficiently examined. Prior research indicates that PL is predominantly linked to student self-regulation and learning outcomes via regulatory mechanisms rather than direct effects (Lim et al., 2020; Moluayonge, 2025). Conceptual and organizational studies suggest that collaborative or shared leadership can facilitate autonomy, accountability, and knowledge sharing—conditions closely associated with self-directed learning (Coun et al., 2019; Hrabalová and Urban, 2024). Despite these overlapping views, there appears to be a lack of empirical studies explicitly modeling collaborative leadership as a mediating mechanism between peer interaction and self-directed learning. This gap points to the need for research that systematically examines collaborative leadership as a process linking peer learning to self-directed learning, particularly in educational contexts.

Theoretically, the relationship between PL and SDL has been addressed in the context of social cognitive theory (Bandura, 1997), self-regulated learning models (Zimmerman and Schunk, 2011), and lifelong learning. Nevertheless, the number of studies systematically examining this relationship quantitatively is relatively low, which reveals a need for more in-depth research on the subject. In their study, Lim et al. (2020) revealed that PL significantly and strongly predicted self-regulated learning strategies (*β* = 0.793). This finding emerged from a study with 347 university students. Pimdee et al. (2023) discovered a robust and positive correlation between PL and SDL in their investigation of 468 pre-service teachers. In a similar vein, Choi and Kim’s (2015) study reported a significant and positive correlation between peer assessment scores and SDL in a higher education context (*r* = 0.254). It is evident that further research is required to test this theorized model and ascertain its relevance to interprofessional collaborative practice. Although measures exist to assess vertical leadership, relational coordination, and leadership practices, there are no established, validated instruments for applying CL to team-based collaborative leadership. It is therefore essential to ascertain whether the constructs of CL accurately measure this particular leadership style. It is conceivable that specific instructional design decisions may facilitate the enhancement of students’ capacity to derive benefit from collaborative, leadership-based changes. This research investigates the role of one such instructional design feature: the timing of collaboration within the PL process.

Within the domain of higher education, students are required to demonstrate aptitude in coping with a multitude of challenges, including but not limited to effective time management, diverse assessment methods, rigorous course content, fostering independence, navigating course challenges, and managing personal characteristics. Moreover, the presence of teaching staff in large classes, coupled with their preoccupation with administrative and non-teaching duties, results in a reduction in their accessibility to students. Such an arrangement has been demonstrated to result in increased segregation and anonymity among student groups, which in turn has a detrimental effect on learning ability, learning attitudes, motivation, and interpersonal relationships (Gannaway, 2022; Hanushek et al., 2003).

In higher education, PL can be considered an effective approach in transitioning students to a new learning paradigm and increasing their interest in deep learning (Hamad et al., 2021). Collaborative PL and SDL environments allow students to manage their learning processes and interact, reconstructing knowledge through peer support, social interaction, and relationships (Guldberg, 2008; Xu et al., 2015).

**Figure 1:**
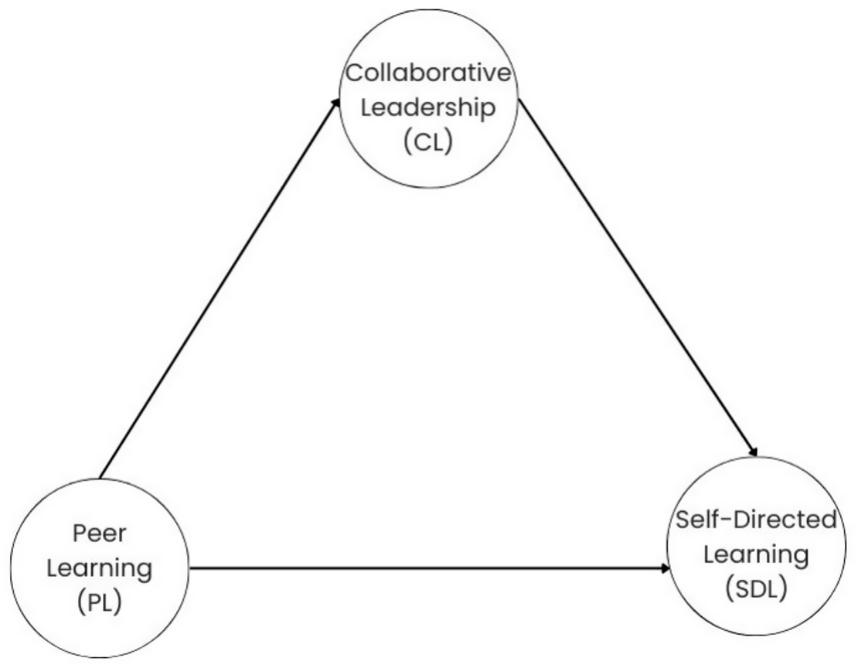
Conceptual framework.

In the context of collaboration and PL, the maximization of human potential is a fundamental priority. Consequently, it has been demonstrated to foster teamwork, cooperation, and quality at every stage of the system (De Hei et al., 2014). Tsai (2013) presents a framework for effective learning methodologies in the context of online learning, which incorporates both collaborative and SDL. In this context, the combination of collaborative and SDL can be regarded as an innovative method for considering students’ expectations. Examining the relationships between PL, CL, and SDL in Türkiye can provide significant external validity to theories developed in the West. This fact is because cultural factors directly shape the functioning and impact of these learning and leadership processes (Bakır and Altunay, 2025; Kunwar, 2021). Türkiye has traditionally had a more hierarchical, collectivist, and rule-bound educational culture (Bakır and Altunay, 2025). Türkiye differs significantly from the individualistic and more horizontal learning environments of the West. Cultural barriers such as resistance to innovation, anti-collaborative attitudes, and the lack of development of professional learning networks in Türkiye differentiate the impact and applicability of PL and CL (Bakır and Altunay, 2025). The results of previous studies suggest that students in Turkish culture are less familiar with peer learning and collaborative leadership practices and are strongly influenced by cultural norms. Findings obtained in Türkiye, by revealing the impact of cultural diversity on learning processes, offer an opportunity to question the universality of Western-centric approaches and develop more inclusive, dynamic models (Bakır and Altunay, 2025; Kunwar, 2021; Lew-Levy et al., 2023). Therefore, studies conducted in the Turkish context may be valuable in testing the cross-cultural validity of our research variables developed in the West and expanding the boundaries of these theories. Examining the interaction of PL, CL, and SDL in Türkiye not only strengthens the external validity of Western theories (Hofstede and Hofstede, 2005) but also offers an original and valuable contribution to understanding the impact of cultural differences on learning and leadership processes. Figure 1 illustrates the theoretical model that has been developed.

## 3 Method

### 3.1 Sample and data collection

In this study, a purposive sampling method was adopted, and the sample was composed of pre-service mathematics and science teachers studying at five different universities in southeastern Türkiye. The main justification for using purposive sampling was to reach individuals who possess the characteristics targeted by the study. Before data collection, the necessary ethical approval was obtained from the Social and Human Sciences Ethics Committee of the affiliated university. The survey, administered in the spring of 2024, consisted of a total of 50 items, including questions on demographic information. Data were collected through face-to-face surveys, and participants were asked to participate in the study voluntarily. In addition, basic demographic characteristics such as participants’ gender, grade level, and project participation status were taken into account to increase sample diversity. 75% (*n* = 408) of the participants were female, and 25% (*n* = 136) were male. When examined in terms of education level, 19.4% (*n* = 106) of the participants were first-year students, 46% (*n* = 250) were second-year students, 19.4% (*n* = 102) were third-year students, and 15.8% (*n* = 86) were fourth-year students. Furthermore, 30.3% (*n* = 165) of the participants reported having participated in a project before, while 69.7% (*n* = 379) stated that they had not participated in any project.

### 3.2 Variables and measures

#### 3.2.1 Self-directed learning scale

The SDL Scale, developed by Lounsbury et al. (2009) and adapted into Turkish by Demircioğlu et al. (2018), was employed in this study. SDL is defined and assessed as a unidimensional construct, aligning with the idea that self-direction encompasses motivation, metacognition, and self-management as a singular overarching trait (Knowles, 1975; Long, 2000). The unidimensional structure aligns with previous validation studies and represents learners’ general capacity to plan, monitor, and regulate their learning. The scale is unidimensional in nature and comprises 10 items. The Cronbach’s alpha coefficient was reported to be 0.85. Confirmatory factor analysis (CFA) results provided adequate evidence for the single-factor structure of the scale. The Cronbach’s alpha coefficient was estimated as 0.955 in this study.

#### 3.2.2 Peer learning scale

The PL Scale is a 12-item scale developed by Kweon and Park (2023) and translated into Turkish by researchers. This scale was used to measure pre-service teachers’ team-based PL levels in this study. The Cronbach’s alpha coefficient for the original scale was determined to be 0.81. Linguistic and conceptual adaptation of the scale was achieved through translation and back-translation processes. Prior to translation, the original scale items were subjected to preliminary review by field experts focusing on conceptual consistency, cultural appropriateness, and linguistic equivalence. To ensure the reliability and validity of the translation process, academic experts in the field of educational leadership oversight (Brislin, 1970) were appointed. The Turkish version of the scale was pilot-tested, and after necessary revisions, the final version was ready for implementation. The items were grouped under three factors: responsibility, initiative, and cooperation, in accordance with the original scale. Responsibility, which represents students’ willingness to share academic and task-related responsibilities; initiative, reflecting proactive engagement and contribution to group processes; and cooperation, capturing mutual support and collaborative interactions among peers. These dimensions are based on the theory of social constructivism and operationalize the behavioral features of effective peer-supported learning. Validity and reliability analyses were then conducted on this final version. CFA demonstrated excellent fit. The Cronbach’s alpha coefficient was estimated as 0.968 in this study.

#### 3.2.3 Collaborative leadership scale

CL Scale, a 28-item, four-dimensional scale developed by Orchard et al. (2020) and translated into Turkish by Gürbüz and Özkul (in press), measured pre-service teachers’ perceptions of interprofessional CL. The CL Scale consists of theoretically grounded subscales: Symbiotic Relationship—mutual awareness of interdependent roles among members; Mindfulness—including mindfulness, situational awareness, and reflective responding within the group; Shared Assets—equitable sharing of tasks, responsibilities, and resources; and Leadership Capacity—the degree to which members exhibit collaborative leadership behaviors. These dimensions reflect the behavioral, relational, and cognitive components of shared leadership. The Cronbach’s alpha coefficient of the original scale was 0.90. CFA showed an excellent fit. In this study, the Cronbach’s alpha coefficients were estimated as 0.951, 0.966, 0.958, and 0.951 for Symbiotic Relationship, Mindfulness, Shared Assets, and Leadership Capacity, respectively.

### 3.3 Data analysis

Structural equation modeling (SEM) was selected as the primary analytical technique because it allows for the explicit modeling of latent constructs while accounting for measurement error at both the indicator and construct levels (Hair et al., 2022). Given that PL, CL and SDL are theoretically multidimensional psychological constructs, SEM provides more accurate and unbiased estimates of structural relationships compared to observed-variable approaches such as multiple regression (Marsh et al., 2020). In addition, SEM allows for the evaluation of global model fit indices (e.g., CFI, TLI, RMSEA, and SRMR), which provide empirical evidence regarding the extent to which the proposed theoretical model adequately represents the observed data (Hair et al., 2022; Hu and Bentler, 1999). This feature is particularly critical for theory-driven studies that aim to test hypothesized relational structures rather than isolated associations. In the present study, SEM made it possible to identify the negligible direct effect of peer learning on self-directed learning alongside the significant indirect effect through collaborative leadership, thereby offering a more nuanced and theoretically meaningful interpretation of the relationships among the constructs. Such an integrated modeling approach would not have been attainable using traditional regression-based methods.

Data analysis followed the two-step approach recommended by Anderson and Gerbing (1988), who emphasized the importance of building reliable measurement models before testing the structural relationships among latent constructs. All analyses were conducted using Mplus Version 8.7 (Muthén and Muthén, 1998–2018). In the first step, CFAs were conducted to validate the measurement models of three latent variables: PL, SDL, and CL. PL and SDL were modeled as unidimensional constructs measured with 12 and 10 items, respectively. CL was modeled as a second-order factor containing four first-order dimensions (symbiotic relationship, mindfulness, shared assets, and leadership capacity) based on 28 observed indicators. Given the ordinal nature of the item responses, all indicators were treated as categorical, and model parameters were estimated using the weighted least squares mean and variance adjusted (WLSMV) estimator, which is suitable for ordinal categorical data and robust to non-normality (Şen, 2020). Prior to conducting the CFA and structural equation model (SEM) analyses, the data were screened for key assumptions. Missing data patterns were evaluated, and cases with missing values on all indicators were excluded listwise, as per WLSMV estimation conventions. The ordinal nature of the data was verified to justify the use of polychoric correlations and categorical specifications. Multicollinearity and outlier checks were also conducted, and no severe violations were observed that would bias the estimation of model parameters. Model fit was evaluated using the comparative fit index (CFI), Tucker–Lewis index (TLI), and standardized root mean square residual (SRMR). Following conventional guidelines (Hu and Bentler, 1999), values of CFI and TLI above 0.90 and SRMR below 0.08 were considered indicative of acceptable model fit. Although root mean square error approximation (RMSEA) values were computed by default in Mplus output, they were not used as primary indicators of fit due to their well-documented sensitivity to degrees of freedom, particularly in models with categorical indicators and few latent variables. In the second step of the analytic approach, a SEM was estimated to examine the hypothesized relationships among PL, CL, and SDL. The model tested whether PL predicted CL and whether both PL and CL predicted SDL. After establishing an acceptable structural model, the analysis was extended to include three covariates: gender (0 = male, 1 = female), grade level (dummy-coded: Grade 1 to Grade 3, with Grade 4 as the reference), and project participation status (0 = no, 1 = yes). These covariates were regressed on the latent variable SDL to account for potential demographic influences. All results were interpreted using standardized path coefficients (STDYX), with statistical significance determined at the *p* < 0.05 level. Effect sizes (i.e., *β* coefficients) were interpreted using Cohen’s (1988) widely adopted benchmarks for small (*β* = 0.10), medium (*β* = 0.30), and large (*β* ≥ 0.50) effects. Model diagnostics, including modification indices and residuals, were reviewed to assess localized areas of misfit and to inform potential model refinements. The composite reliability coefficient was calculated to determine the internal consistency of each factor analyzed.

## 4 Results

To evaluate the measurement properties of the instruments, three CFAs were conducted for PL, SDL, and CL. The fit indices of the CFA models are presented in Table 1. The one-dimensional model for PL, consisting of 12 items, demonstrated acceptable model fit, with SRMR = 0.026, CFI = 0.977, and TLI = 0.972. Similarly, the 10-item SDL scale showed a satisfactory model fit (SRMR = 0.023, CFI = 0.979, TLI = 0.973), supporting the single-factor solution of the SDL construct. Composite reliability coefficients were estimated as 0.969 and 0.978 for SDL and PL, respectively. The CL construct, modeled as a second-order factor comprising symbiotic relationship, mindfulness, shared assets, and capacity to lead (28 items in total), yielded strong model fit indices (SRMR = 0.022, CFI = 0.980, TLI = 0.978). Composite reliability coefficients were estimated as 0.968, 0.977, 0.972, and 0.968 for symbiotic relationship, mindfulness, shared assets, and leadership capacity, respectively. These results indicate the adequacy of the proposed factor structures for subsequent structural modeling. A full structural equation model was then estimated to test the hypothesized relations among PL, CL, and SDL. The fit indices of this model are reported in Table 1. As shown in Table 1, the full structural equation model showed good model fit (SRMR = 0.027, CFI = 0.976, TLI = 0.975). Another full structural equation model was also estimated, specified to examine the interrelations among PL, CL, and SDL, including covariates: gender (0 = male, 1 = female), grade level (dummy-coded: Grade 1 to Grade 4), and project participation status (0 = no, 1 = yes). The final model demonstrated excellent fit to the data, with SRMR = 0.060, CFI = 0.980, and TLI = 0.979, meeting widely accepted thresholds for model adequacy in SEM (e.g., Hu and Bentler, 1999).

**Table 1:** Model fit statistics for SEM.

Variables	*χ* ^2^	df	RMSEA [90% CI]	SRMR	CFI	TLI
PL	803.264*	54	0.160 [0.150, 0.170]	0.026	0.977	0.972
SDL	372.346*	35	0.134 [0.122, 0.146]	0.023	0.979	0.973
CL	1797.186*	344	0.089 [0.085, 0.093]	0.022	0.980	0.978
Full SEM	3302.621*	1,168	0.058 [0.056, 0.060]	0.027	0.976	0.975
Full SEM with covariates	3135.670*	1,413	0.047 [0.045, 0.050]	0.060	0.980	0.979

^*^p < 0.001.

**Figure 2:**
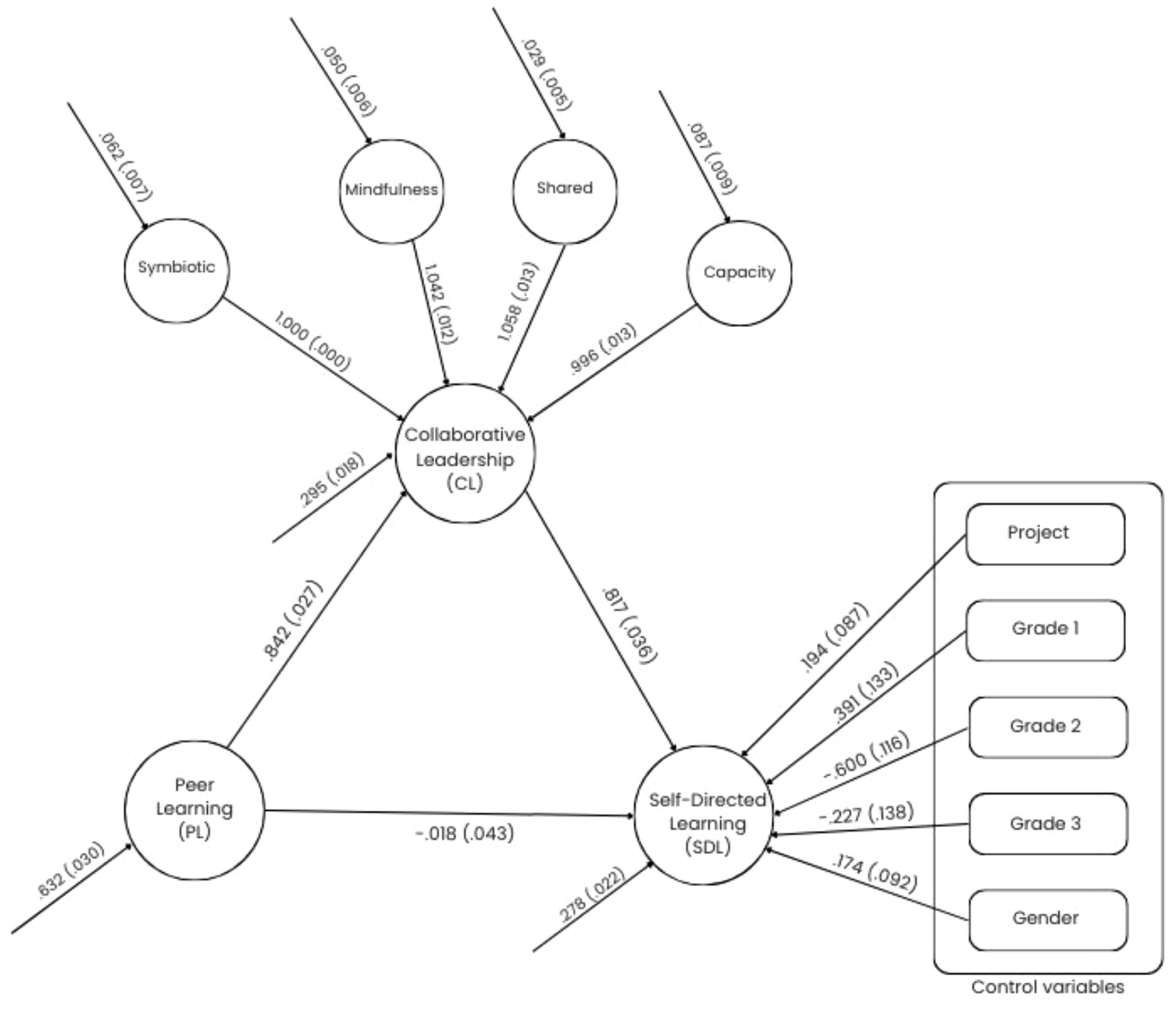
Path diagram for final SEM with unstandardized coefficients.

The SEM results revealed that PL had a strong and statistically significant effect on CL (*β* = 0.776), which in turn significantly predicted SDL (*β* = 0.777). Both represent large effect sizes according to Cohen’s (1988) conventions. These findings support the hypothesized sequential mediation model, in which students’ engagement in peer-based learning fosters collaborative relationship skills, which subsequently enhance their self-directed learning behaviors (see Figure 2).

Standardized path coefficients for the relationship between covariates and SDL are presented in Table 2. Unstandardized path coefficients are also presented in Figure 1. As shown in Table 2, covariate analysis revealed that Grade level effects were most pronounced for Grade 1 and Grade 2 students, who demonstrated significantly lower levels of SDL compared to their Grade 4 peers (e.g., Grade1 → SDL: *β* = −0.171, *p* < 0.003; Grade2 → SDL: *β* = −0.330, *p* < 0.001). Moreover, project participation status was found to be a statistically significant and positive predictor of SDL (Project → SDL: *β* = 0.098, *p* = 0.025), suggesting that students engaged in structured projects may benefit from self-directed learning. *R*^2^ values were estimated as 0.662, 0.923, 0.942, 0.966, 0.894, and 0.603 for SDL, symbiotic relationship, mindfulness, shared assets, capacity to lead, and CL latent variables, respectively. The structural model demonstrated strong explanatory power for the key latent variables (Cohen, 1988; Kline, 2023). Specifically, it accounted for 66.2% of the variance in SDL and 60.3% of the variance in the higher-order CL construct. For the four first-order dimensions of CL, the explained variances were 92.3% for symbiotic relationship, 94.2% for mindfulness, 96.6% for shared assets, and 89.4% for capacity to lead.

**Table 2:** Path coefficients for the relationship between covariates and SDL.

Variable	B	SDL	*p*
		S.E.	
PL → SDL	−0.016	0.037	0.674
CL → SDL	0.777	0.034	**<0.001**
CL → PL	0.776	0.017	**<0.001**
Gender → SDL	−0.083	0.044	0.058
Grade1 → SDL	−0.171	0.057	**0.003**
Grade2 → SDL	−0.330	0.062	**<0.001**
Grade3 → SDL	−0.098	0.059	0.100
Project → SDL	0.098	0.044	**0.025**

SDL, Self-Directed Learning; PL, Peer Learning; CL, Collaborative Leadership. Bold values indicate statistical significance *p* < 0.05.

## 5 Discussion

The present study was conducted with mathematics and science teacher education students from Southeastern Türkiye who had experience preparing projects in education. The present study examined the relationship between PL and SDL, the mediating role of CL, and the effects of demographic variables using SEM.

Contrary to some previous findings, the present study shows that PL does not have a statistically significant direct effect on SDL when CL is included in the model (*β* = −0.016, *p* = 0.674). This finding suggests that, within the current sample, peer interaction alone is not sufficient to promote SDL behaviors unless it is accompanied by effective CL processes. This result partially contrasts with earlier studies reporting a positive association between PL and SDL or closely related constructs such as self-regulated learning. For instance, Lim et al. (2020) found that PL strongly predicted SDL strategies (*β* = 0.793), while Pimdee et al. (2023) reported a robust positive relationship between PL and SDL among pre-service teachers. However, these studies primarily examined direct relationships or employed models that did not account for leadership-related mechanisms within peer groups. The present findings extend this literature by demonstrating that the direct effect of PL on SDL may diminish once the social-organizational structure of peer interaction, operationalized here as CL, is taken into account.

In contrast, CL emerges as a strong and statistically significant predictor of SDL (*β* = 0.777, *p* < 0.001). This result highlights the central role of leadership processes in shaping students’ capacity to manage, regulate, and take responsibility for their learning. Consistent with prior research, CL appears to foster trust, shared responsibility, and autonomy-supportive interactions, which are key conditions for the development of SDL (Hrabalová and Urban, 2024; Xie et al., 2019). Studies focusing on team-based and collaborative learning environments similarly emphasize that leadership distributed among group members enhances motivation, engagement, and regulatory behaviors (De Hei et al., 2014; Tsai, 2013; Wu et al., 2025). The strong effect observed in this study suggests that SDL is more likely to emerge not merely from peer interaction itself, but from how such interaction is organized, guided, and shared within the group.

Furthermore, the model reveals a high and statistically significant relationship between PL and CL (*β* = 0.776, *p* = 0.017), indicating that PL contexts are closely associated with the development of CL practices. This finding aligns with previous studies suggesting that learning communities characterized by active peer engagement tend to promote shared leadership roles, mutual influence, and collective responsibility (Yilmaz et al., 2020; Alkhawaldeh and Khasawneh, 2023). In these environments, a single individual does not concentrate leadership; instead, it emerges through interaction, dialogue, and joint problem-solving, thereby reinforcing both collaboration and learning quality.

Taken together, these findings support a mediation-based interpretation of the relationship between PL and SDL. While PL creates opportunities for interaction and knowledge exchange, its impact on SDL appears to operate primarily through CL processes rather than through a direct pathway. This pattern is consistent with theoretical perspectives emphasizing socially embedded regulation and shared responsibility as precursors to individual self-direction in learning (Van Woezik et al., 2021). Unlike previous mediation studies that have primarily positioned self-regulated learning as the intervening mechanism (Lim et al., 2020; Moluayonge, 2025), the present study contributes to the literature by empirically demonstrating the mediating role of CL in an educational context.

The results of the study findings indicate that demographic variables such as grade level and project experience (excluding gender) create significant differences in students’ SDL and CL variables. Students with project experience, like the literature, benefit more from PL and leadership processes (Lim et al., 2020; Wu et al., 2025). This may be due to the natural peer-learning nature of the process of gaining project experience. Another issue is that leadership processes emerge unintentionally in project partnerships. Structural equation modeling analyses in this study also demonstrate the natural relationship between these two variables. Our research results, which also differ by grade level, indicate that students’ SDL skills and leadership competencies increase throughout their academic development. These findings emphasize the importance of instructional designs based on student characteristics and the need to consider individual differences in the learning process (Lawson and Mayer, 2023). PL processes among pre-service teachers during the project development process fostered openness to idea exploration, which in turn enhanced critical thinking and the SDL of other group members. PL and argumentative conversations can be important drivers of self-directed learning in teams. Their impact is clear when designing collaborative learning for future professionals.

## 6 Conclusion

This study indicates that PL and CL together explain approximately 66% of the total variation in SDL, controlling for various school and student variables such as grade level, gender, and project experience. While SDL has been demonstrated to be influenced by a variety of factors, PL has been shown to increase students’ interaction with their environment, cognitive development, and social interaction. The present study confirms, as expected, that CL significantly influences this relationship. Therefore, we recommend developing CL by promoting PL in schools. Research demonstrates that these practices foster a conducive environment for CL, strengthening educators’ belief in their ability to impact student learning. The present study posits that CL appears to support the other two variables, particularly in a collective work environment where PL is active. These findings imply several significant implications. Upper management predominantly associates with leadership in numerous non-Western contexts, and issues such as student leadership receive inadequate attention. Nevertheless, the findings of this study demonstrate that student leadership can emerge even in situations where formal leadership mechanisms are not available for students. Research has demonstrated that the implementation of student leadership in these contexts yields specific benefits for both peers and organizational processes. We cannot overstate the importance of PL and the natural development of CL positions among team members during the project development process. Another variable that facilitates a deeper understanding of this process is SDL. It is imperative to develop a more profound comprehension of the natural roles (e.g., leadership) that are anticipated to emerge among team members who are united by a shared purpose and the mechanisms through which these roles are established. It is therefore recommended that policymakers and educational administrators provide support for student interactions and empower them to act as leaders. The findings of this study demonstrate the significance of the correlation between PL and CL, emphasizing the value of school-specific project-based activities in fostering the development of student leadership within such educational environments. The systematic promotion of PL practices and the fostering of CL within educational processes have been identified as potentially efficacious strategies for the development of students’ SDL skills.

## 7 Limitations and future directions of the study

First, the impact of PL on CL and SDL was examined at a single level. For a more reliable investigation, it would be beneficial for future studies to employ multilevel analyses that account for the nested structure of school data. Second, the cross-sectional nature of this study makes it difficult to draw definitive conclusions about the causal direction of the relationships between variables. Future research would benefit from using more robust research designs that allow for causal inference, such as longitudinal studies. Third, data were collected through self-reporting. Future research could consider the views of different stakeholder groups (academics, etc.). Finally, the augmentation of project-based work in educational institutions, coupled with the meticulous design of learning environments that inherently nurture leadership skills in students, has the potential to fortify both the collective learning culture and the leadership and learning competencies of pre-service teachers.

## Data Availability

The raw data supporting the conclusions of this article will be made available by the authors, without undue reservation.
